# LINC00963 targeting miR‐128‐3p promotes acute kidney injury process by activating JAK2/STAT1 pathway

**DOI:** 10.1111/jcmm.15211

**Published:** 2020-04-09

**Authors:** Li‐Bo Xie, Bo Chen, Xue Liao, Yi‐Feng Chen, Rui Yang, Si‐Rong He, Li‐Jun Pei, Rui Jiang

**Affiliations:** ^1^ Department of Urology Nephropathy Clinical Medical Research Center of Sichuan Province The Affiliated Hospital of Southwest Medical University Luzhou China; ^2^ Department of Human Anatomy School of Basic Medical Sciences Southwest Medical University Luzhou China; ^3^ College of Clinical Medicine Southwest Medical University School of Clinical Medical Sciences Luzhou China; ^4^ Department of Immunology School of Basic Medical Sciences Chongqing Medical University Chongqing China

**Keywords:** acute kidney injury, apoptosis, LINC00963, miR‐128‐3p, STAT1 pathway

## Abstract

The role of long non‐coding RNAs (lncRNAs) in kidney diseases has been gradually discovered in recent years. LINC00963, as an lncRNA, was found to be involved in chronic renal failure. However, the role and molecular mechanisms of LINC00963 engaged in acute kidney injury (AKI) were still unclear. In this study, we established rat AKI models by ischaemia and reperfusion (I/R) treatment. Urea and creatinine levels were determined, and histological features of kidney tissues were examined following HE staining. CCK8 assay was chosen to assess the viability of hypoxia‐induced HK‐2 cells. Dual‐luciferase reporter gene assays were performed to verify the target relationship between LINC00963 and microRNA. The mRNA and protein levels were assayed by RT‐qPCR and Western blot, respectively. Annexin V‐FITC/PI and TUNEL staining were used to evaluate apoptosis. LINC00963 was highly expressed in the cell and rat models, and miR‐128‐3p was predicted and then verified as a target gene of LINC00963. Knockdown of LINC00963 reduced acute renal injury both in vitro and in vivo. LINC00963 activated the JAK2/STAT1 pathway to aggravate renal I/R injury. LINC00963 could target miR‐128‐3p to reduce G1 arrest and apoptosis through JAK2/STAT1 pathway to promote the progression of AKI.

## 1 INTRODUCTION

Acute kidney injury (AKI) is a common clinical symptom often characterized by the abrupt loss of normal kidney functions, leading to the increase of morbidity and mortality.^1^ AKI could be induced by various conditions, including kidney ischaemia, exposure to toxic substances, obstruction of urinary tracts and severe inflammation.^2^ AKI is associated with a high mortality, great economic and social burdens, particularly in critically ill cases.^3^ Numerous severe complications are also associated with AKI, such as metabolic acidosis, body fluid imbalance, uraemia and chronic kidney disease. Due to the lack of an effective therapeutic reagent, pathogeny expelling and renal replacement therapy remain the major treatment strategies at present.^4^ Renal ischaemia‐reperfusion (I/R) injury has been proved to be the major cause of AKI. In spite of several treatments to relieve clinical symptoms, there is still no effective treatment for AKI. Therefore, it is important to explore more therapeutic strategies for AKI.

Long non‐coding RNAs (lncRNAs) are transcripts of more than 200 nucleotides, which have limited or no protein‐coding potential. Emerging evidence found that they can regulate the growth of tumours,^5^ and it remains unclear that lncRNAs are involved in AKI progress. LINC00963 (long intergenic non‐protein‐coding RNA 963) is an RNA gene and is affiliated with the non‐coding RNA class. Diseases involved in LINC00963 included prostate disease and non‐papillary renal cell carcinoma. Linc00963 was considered as a regulatory factor of cell apoptosis. Wang et al have found that Linc00963 in C4‐2 cells (hormone‐insensitive prostate cell line) could increase cell viability and reduce apoptosis.^6^ Previous studies showed that LINC00963 was found to be involved in chronic renal failure.^7^ However, the molecular mechanisms of LINC00963 engaged in acute kidney injury (AKI) still need to be explored. Therefore, in this study, the hypoxia‐induced HK‐2 cell injury model and rat I/R injury model were established to investigate the role of LINC00963 in AKI. The results may provide a reference for the clinical development of a new and effective treatment method for AKI.

## 2 MATERIALS AND METHODS

### 2.1 Cell culture, hypoxia‐induced injury model, transfection and proliferation

HK‐2 cells were cultured in DMEM/Ham's F12 (50%/50%, vol/vol) supplemented with 10%FBS (Gibco, Life Technologies^™^), 100 U/mL penicillin and 100 μg/mL streptomycin under normoxic conditions at 37°C. Hypoxia‐induced cell injury model was established by using three gas incubators. Specifically, HK‐2 cells were firstly implanted in six‐well plates filled with above‐mentioned culture media under anoxic environment (94% N_2_/1% O_2_/5% CO_2_). After 24 hours, fresh medium was changed and the cells were transferred to aerobic environment (95% air/5% CO_2_) for 12 hours. HK‐2 cells were divided into different groups according to the time of detection and the type of interference agents. RNA interference was made by using Lipofectamine 2000 (Invitrogen) transfection according to manufacturer's instructions. CCK8 Kit (Solarbio, China) was used for the test of cell viability in multiwall plate reader (BioTek Instruments, Inc). Annexin V‐FITC/PI Apoptosis Detection Kit (Yeasen, China) was used to evaluate apoptosis of each group by flow cytometry.

### 2.2 Establishment of AKI model

Male Sprague Dawley (SD) rats aged between 6 and 8 weeks and weighing 200‐300 g were kept at (25 ± 3)°C with a 12‐hour light‐dark cycle. All the experiments were approved through institutional ethics committee (Approval number: 20180306096). To induce AKI model, rats in IR groups were subjected to I/R surgery as previously described.^8^ Briefly, a median abdominal incision was made to expose the bilateral kidneys in retroperitoneal space after being generally anesthetized with isoflurane. The right nephrectomy was performed for all the experiment rats. Left renal pedicle of each rat was then mobilized and occluded with a microvascular clamp for 40 minutes, followed with reperfusion by removal of the clamp for 6, 12 and 24 hours. Rats in sham group were subjected to similar experimental operations except for the occlusion of renal pedicle. All rats were finally killed via the previous incision under deep anaesthesia with isoflurane, and then, the tissue of left kidney was collected for the following assays. In the intervene experiment group, si‐LINC00963 (interference fragments of LINC00963, designed by Shanghai Gene Chem Company, China) and its downstream molecule inhibitor were given to the left kidney by multi‐local intraparenchymal injection described by Hao et al ^9^ before occluding the renal pedicles.

### 2.3 Renal function and histopathological examination

Blood samples were collected and then centrifuged at room temperature. Blood urea nitrogen (BUN) and serum creatinine (Scr) levels in the serum were analysed by alkaline picrate method with Roche Modular P800 Analyzer (Roche, China). Portions of the left kidney tissues were fixed in 10% formalin. After gradient alcohol dehydration, the tissues were embedded in paraffin wax. Five‐μm‐thick sections were prepared and stained with haematoxylin and eosin（HE）. Every sample was randomly cut into five histological sections. Histopathology examination was completed by using Nikon eclipse TE2000‐U Microscope.

### 2.4 RNA Fluorescent in Situ Hybridization (FISH) assay

DIG‐labelled LINC00963 probes were designed and synthesized by RiboBio as previously described.^10^ Briefly, paraffin sections were dewaxed with an xylene and rehydrated with a gradient alcohol, then digested with protease K (20 µg/mL) at 37°C for 25 minutes. After washing with PBS, 3% methanol‐H_2_O_2_ was used to block endogenous peroxidase, and the prehybridization solution was added to the mixture and incubated at 37°C for 1 hour. After incubation, sections were poured the prehybridization solution, dropped the hybridization solution containing LINC00963 probe at a concentration of 6 ng/µL and hybridized at 37°C in the incubator overnight. After hybridization, it was washed and sealed, and then, anti‐digoxin labelled peroxidase (anti‐DIG‐HRP) was added to incubate at 37°C for 50 minutes, then wash three times with PBS and add FITC‐TSA. Nuclei were stained with DAPI.

### 2.5 Dual‐Luciferase reporter assay

The target spot of LINC00963 was identified by RegRNA 2.0 before investigating the correlation between LINC00963 and miR128‐3p by luciferase reporter assays. MiR128‐3p mimics (Tongyong, China) or control mimics were transfected into HK‐2 cells by Lipofectamine 2000 (Invitrogen). After 48 hours of transfection, the activity of luciferase reporter gene was detected by double luciferase detection system.

### 2.6 Pull‐down assay

RNA pull‐down assay was performed by Pierce Magnetic RNA‐Protein Pull‐Down Kit (Thermo Scientific, USA), according to manufacturer's instructions. (NC‐biotin: 5´‐CCUGGUUUUUAAGGAGUGUCGCCAGAGUGCCGCGAAUGAAAAA‐3´,LINC00963‐biotin: 5´‐TTTTTAGTAGAGACGGGGTTTCACTGTGTTAGCC3´).

### 2.7 Real‐time quantified PCR (qRT‐PCR)

Total RNA was isolated with TRIzol reagent (Takara, Japan) and was reversely transcribed to cDNA, and PCR amplification was carried out according to the manufacturer's instructions. The results were analysed by the biological system 7300 fast real‐time PCR system.

### 2.8 Western blot assay

Proteins were extracted from cells and renal tissues by using protein extraction kit (Bio‐Rad, USA). The protein extract (20‐100 μg) was loaded on SDS‐PAGE for separation of proteins. Proteins were transferred to a polyvinylidene fluoride (PVDF) membrane (Millipore) and then incubated with anti‐GAPDH (Abcam), or Bax, Bcl‐2, CytC, CDK2, CyclinE (Proteintech) and JAK2, p‐JAK2, STAT1 and p‐STAT1 (Santa Cruz Biotechnology) antibodies and the corresponding secondary antibodies. Protein was visualized by enhanced chemiluminescence (ECL) (GE Healthcare). Western blot was analysed by QuantityOne software (Bio‐Rad).

### 2.9 Statistical analysis

Statistical analyses were performed using GraphPad Prism 5. All data in this study were expressed as the mean ± standard error means (SEM) from at least three independent experiments. The one‐way variance analysis and Duncan multiple range test were performed for the group comparison. All comparisons with a *P* < .05 were considered statistically significant.

## 3 RESULTS

### 3.1 LINC00963 is overexpressed in hypoxia‐induced HK‐2 cells and renal I/R rat

**FIGURE 1:**
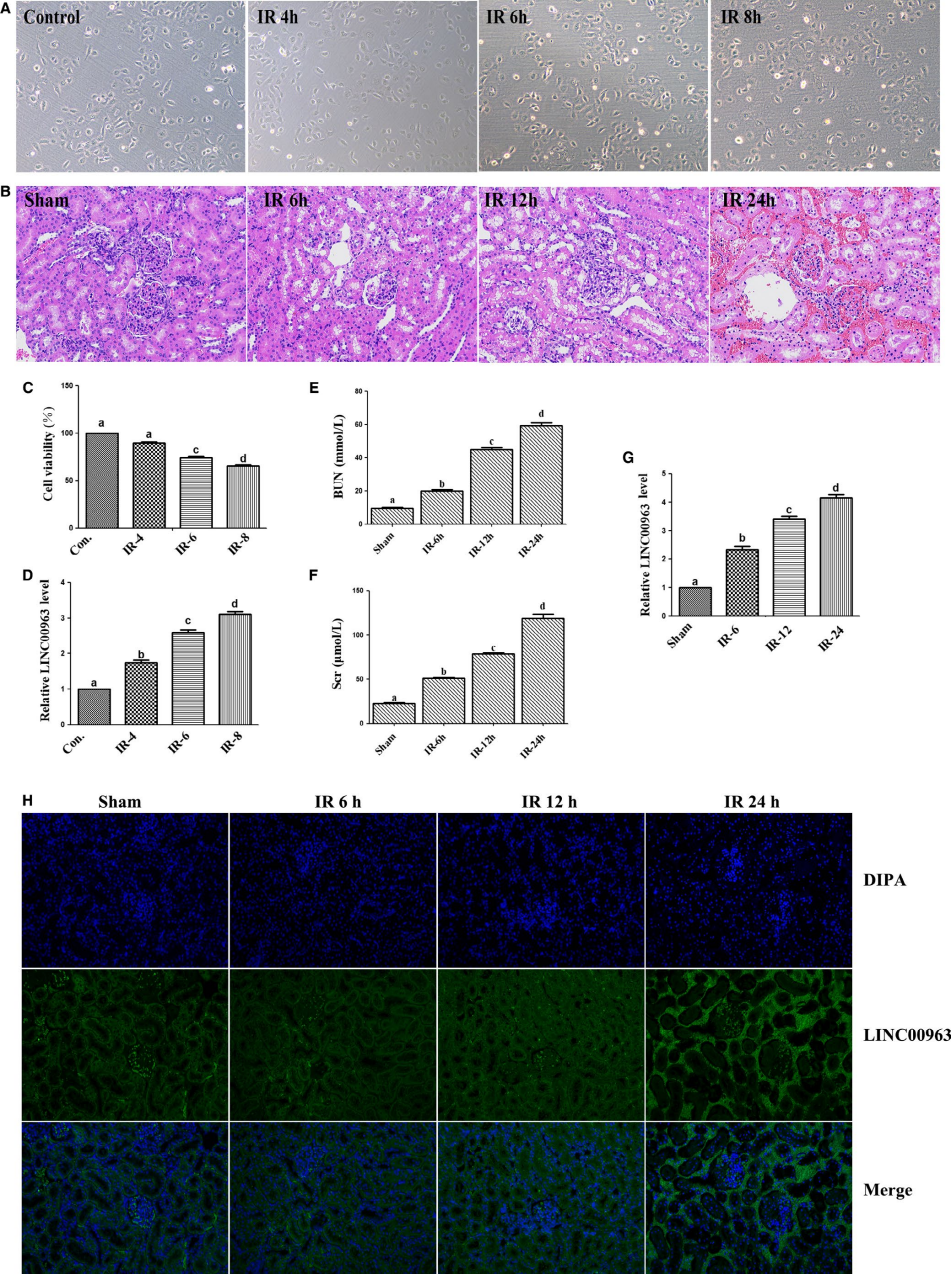
Expression of LINC00963 in hypoxia‐induced HK‐2 cells and AKI rat models. A, Representative morphology of HK‐2 cell models. B, HE staining of renal tissues in AKI rat models. C, The viability of HK‐2 cells analysed by CCK‐8. D, Relative LINC00963 mRNA expression in HK‐2 cell models at 4, 6 and 8 h of reoxygenation. (E) BUN and (F) Scr levels of AKI model rats after reperfusion at various time‐points. G, Relative LINC00963 mRNA expression of LINC00963 in renal tissue. H, RNA FISH analysis of LINC00963 in hypoxia‐induced HK‐2 cells after reoxygenation for 8 h. Data were expressed as mean ± SEM, and different letters represented significant differences (*P* < .05) between groups

Hypoxia‐induced model of HK‐2 cells and I/R‐induced rat AKI model were established to determine whether LINC00963 was involved in renal I/R injury. The success of cell and rat models was firstly evaluated by observing the morphology (Figure 1A) and pathological features (Figure 1B) at different time‐point. CCK8 assay indicated that the viability of HK‐2 cells gradually decreased as the reoxygenation duration from 4 to 8 hours, and the difference was statistically significant between these time‐points, except for the comparison between 0 and 4 hours (Figure 1C). The results of HE staining showed that the morphological structure of renal tissues was normal in the sham group with no changes, while the renal tissues from 6 to 24 hours after reperfusion in the IR groups showed the loss of brush borders, vacuolar degeneration and necrosis in the epithelial cells, as well as dilatation, cast formation and cell debris in the kidney tubules. The BUN and Scr levels in the IR groups (IR‐6, IR‐12, IR‐24) were significantly higher than sham group (Figure 1E,F).

The expression of INC00963 in cells and renal tissues was determined by qRT‐PCR. It was found that LINC0096 mRNA was aberrantly up‐regulated in HK‐2 cells of hypoxia‐induced group after reoxygenation compared to that in control group (Figure 1D), as well as in AKI rat model (Figure 1G). Furthermore, the RNA FISH assay revealed that LINC0096 was mainly located in the cytoplasm (Figure 1H).

### 3.2 MiR‐128‐3p is the target of LINC00963 in HK‐2 cells

**FIGURE 2:**
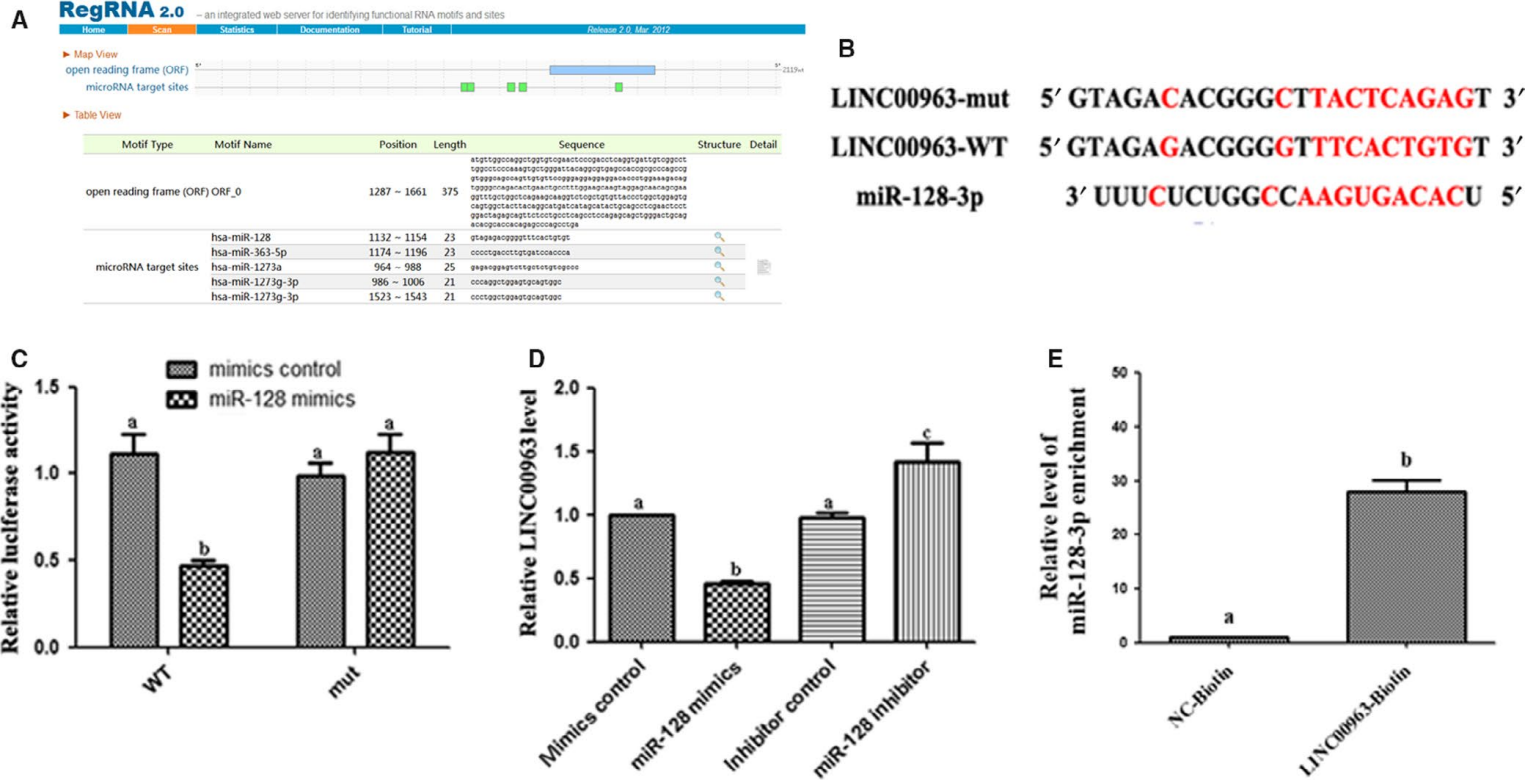
Targeting relationship between miR‐128‐3p and LINC00963. A, Potential binding sites of LINC00963 and miR‐128‐3p via RegRNA 2.0. B, Potential binding sites between LINC00963 and miR‐128‐3p. C, Binding relationship of LINC00963 and miR‐128‐3p was confirmed by dual‐luciferase reporter gene assay. D, Relative mRNA expression of LINC00963 in HK‐2 cells after transfected with miR‐128‐3p mimics/inhibitor. E, RNA pull‐down assay and qPCR detection of LINC00963 binding to miR‐128‐3p. Data are expressed as mean ± SEM, and different letters represented significant differences (*P* < .05) between groups

The target of LINC00963 was identified by RegRNA 2.0, and the search results are shown in Figure 2A. There were potential binding sites at the 3’‐ and 5’‐UTR among the sequence in LINC00963 and miR‐128‐3p (Figure 2B). To validate this bioinformatics predication, dual‐luciferase reporter gene assay was carried out to indicated that LINC00963‐WT presented lower luciferase activity than the corresponding MUT group, confirming the binding relationship of LINC00963 and miR‐128‐3p (Figure 2C). Then, PCR analysis showed that down‐regulation of miR‐128‐3p significantly up‐regulated the mRNA level of LINC00963 mRNA. Conversely, overexpression of miR‐128‐3p decreased the LINC00963 mRNA level (Figure 2D). Additionally, the RNA pull‐down assay and qPCR showed that LINC00963 was capable of interacting with miR‐128‐3p (Figure 2E). These findings suggested that LINC00963 targeted miR‐128‐3p.

### 3.3 miR‐128‐3p‐ASO restrained the G1 arrest and apoptosis suppression of si‐LINC00963 in hypoxia‐induced HK‐2 cells

**FIGURE 3:**
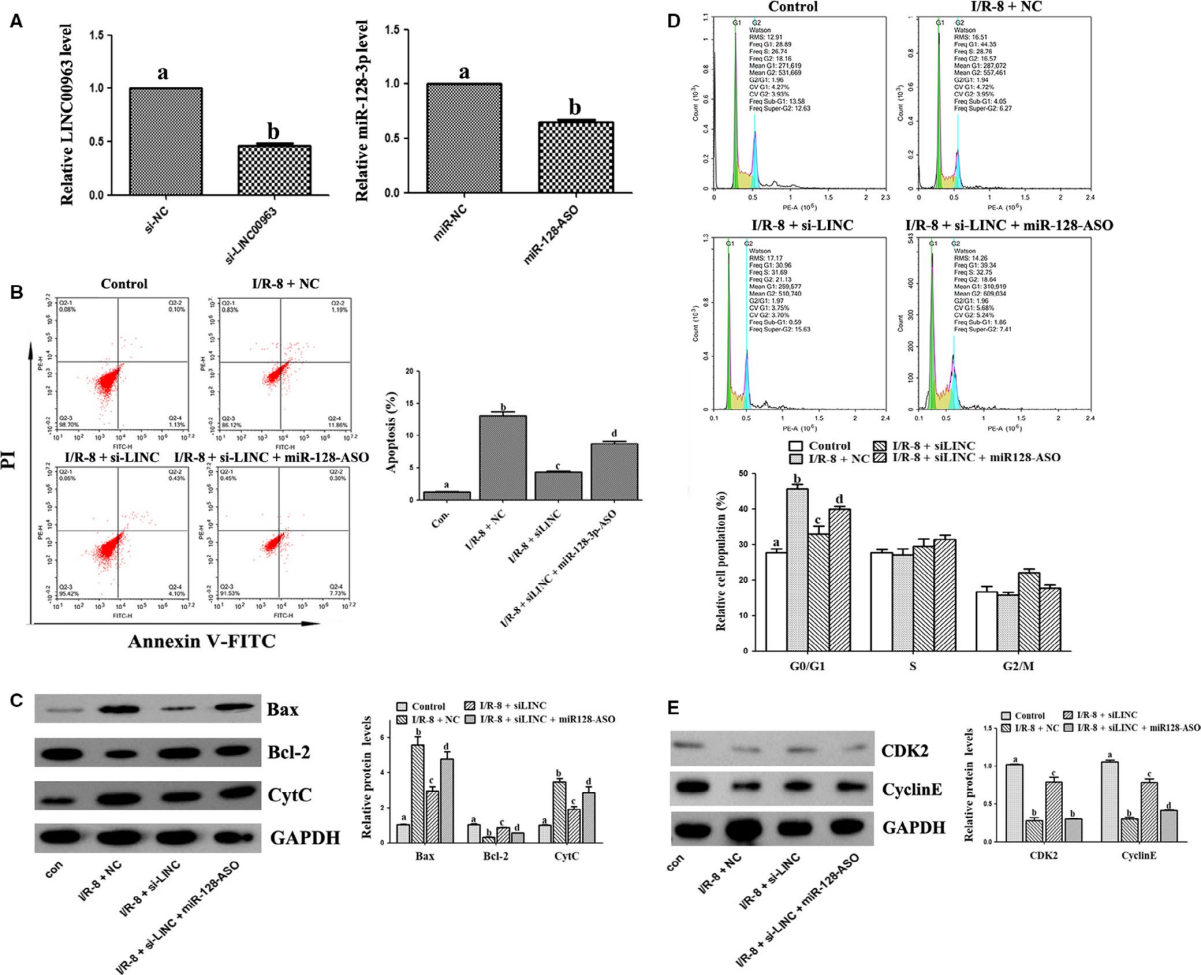
The impacts of LINC00963 and miR‐128‐3p on the apoptosis and cell cycle in hypoxia‐induced HK‐2 cells. A, Cell transfection efficiency of si‐LINC00963 and miR‐128‐3p‐ASO. B, Apoptosis analysis examined by flow cytometric using Annexin V/PI assay. C, Expression of apoptosis‐related proteins by Western blot. D, The distribution of cell cycle analysed by flow cytometry. E, Expression of cell cycle‐related proteins by Western blot. Data are expressed as mean ± SEM, and different letters represent significant differences (*P* < .05) between groups

RT‐PCR was used to detect the relative mRNA level of LINC00963 and miR‐128‐3p after each of them been knocked down in HK‐2 cells, separately. As shown in Figure 3A, LINC00963 mRNA in HK‐2 cells of si‐LINC00963 group was significantly lower than that in the si‐NC group, and the expression of miR‐128‐3p mRNA was also significantly lower after miR‐128‐3p‐ASO administration. Flow cytometry detection (Figure 3B) showed that miR‐128‐3p‐ASO could partially reverse the effects of LINC00963 knockdown on cell apoptosis in hypoxia‐induced HK‐2 cells. Next, the level of apoptosis‐related proteins was detected. Figure 3D shows that knockdown of LINC00963 could significantly reduce the expression of apoptotic proteins (Bax and CytC) induced by hypoxia. However, transfection of miR‐128‐3p‐ASO could synchronously increase the expression of Bax and CytC again. The expression trend of anti‐apoptotic protein Bcl‐2 was contrary to that of Bax. The block of LINC00963 and miR‐128‐3p also resulted in the changes of cell cycle of HK‐2 cells. As shown in Figure 3D, G1 phase arrest was induced by hypoxia. Knockdown of LINC00963 significantly decreased G1 phase arrest, and transfection of miR‐128‐3p‐ASO increased G1 phase arrest. The expression of cell cycle regulatory proteins (CyclinE and CDK2) further confirmed the above results (Figure 3E).

### 3.4 Knockdown of LINC00963 reduced renal injury in rats

**FIGURE 4:**
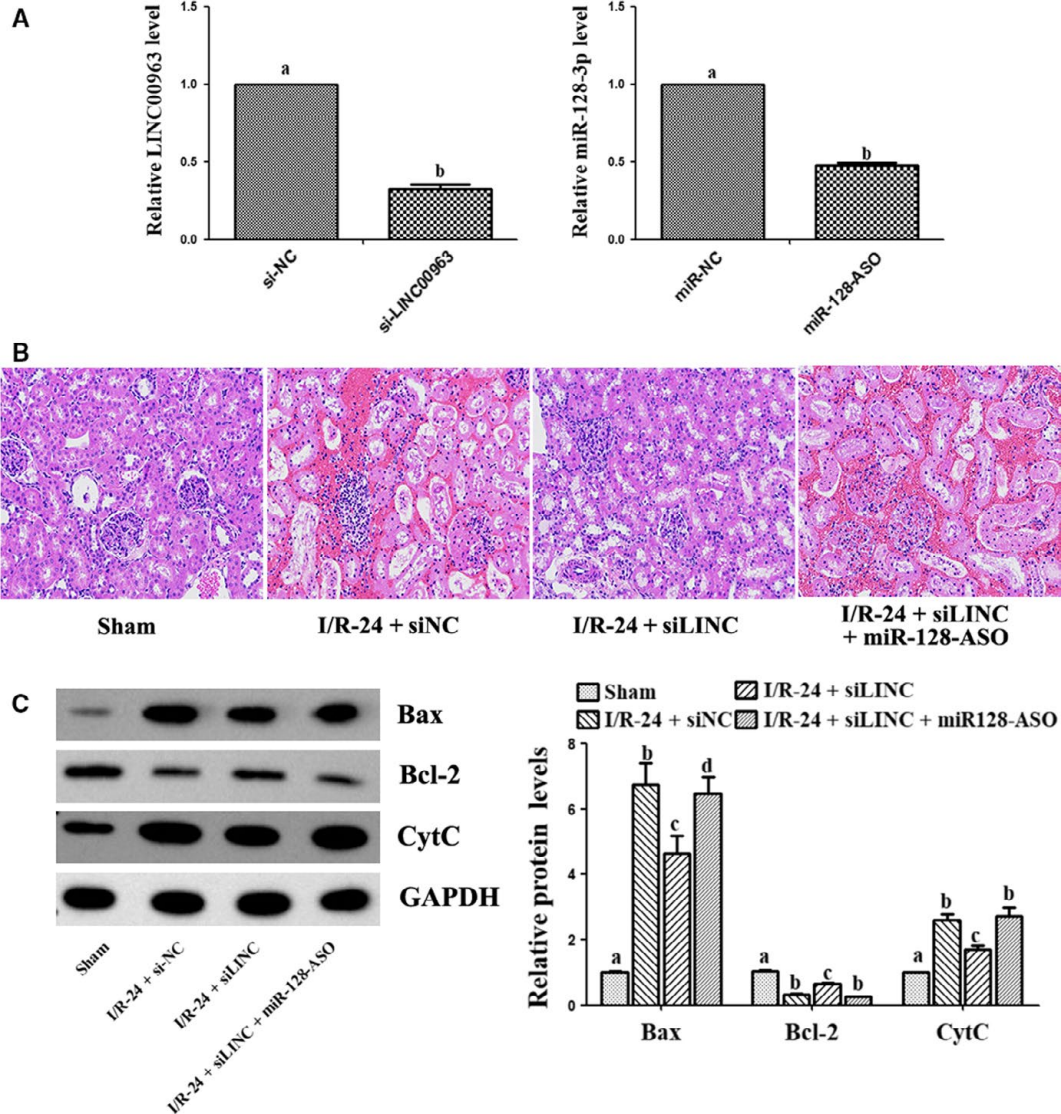
Effect of si‐LINC00963 on renal I/R‐induced AKI rat model. A, Transfection efficiency of si‐LINC00963 and miR‐128‐3p‐ASO in renal tissues by qRT‐PCR. B, HE staining of I/R renal after reperfusion for 24 h among groups. C, Expression of apoptosis‐related proteins by Western blot. Data are expressed as mean ± SEM, and different letters represent significant differences (*P* < .05) between groups

The effect of LINC00963 was further investigated in rat AKI model by si‐LINC00963 plasmid (si‐LINC). Renal tissue qRT‐PCR showed that compared with the negative control, si‐LINC and miR‐128‐3p‐ASO groups showed markedly reduction in the expression of LINC00963 and miR‐128‐3p, respectively (Figure 4A). HE staining showed no abnormalities in the sham group. The renal tubules in the I/R‐24 group were significantly dilated, with renal interstitial hyperaemia, oedema and inflammatory cell infiltration. These injuries were significantly decreased in the I/R‐24+si‐LINC group; however, in I/R‐24+si‐LINC+miR‐128‐ASO group, the damage aggravated again (Figure 4B). Western blot of renal tissues showed that silence of LINC00963 significantly reduced the expression of Bax and CytC induced by I/R injury, and transfection of miR‐128‐3p‐ASO increased Bax and CytC again. However, the changes of Bcl‐2 were opposite to that of Bax (Figure 4C).

### 3.5 LINC00963 activated JAK2/STAT1 pathway to promote apoptosis in hypoxia‐induced HK‐2 cells

**FIGURE 5:**
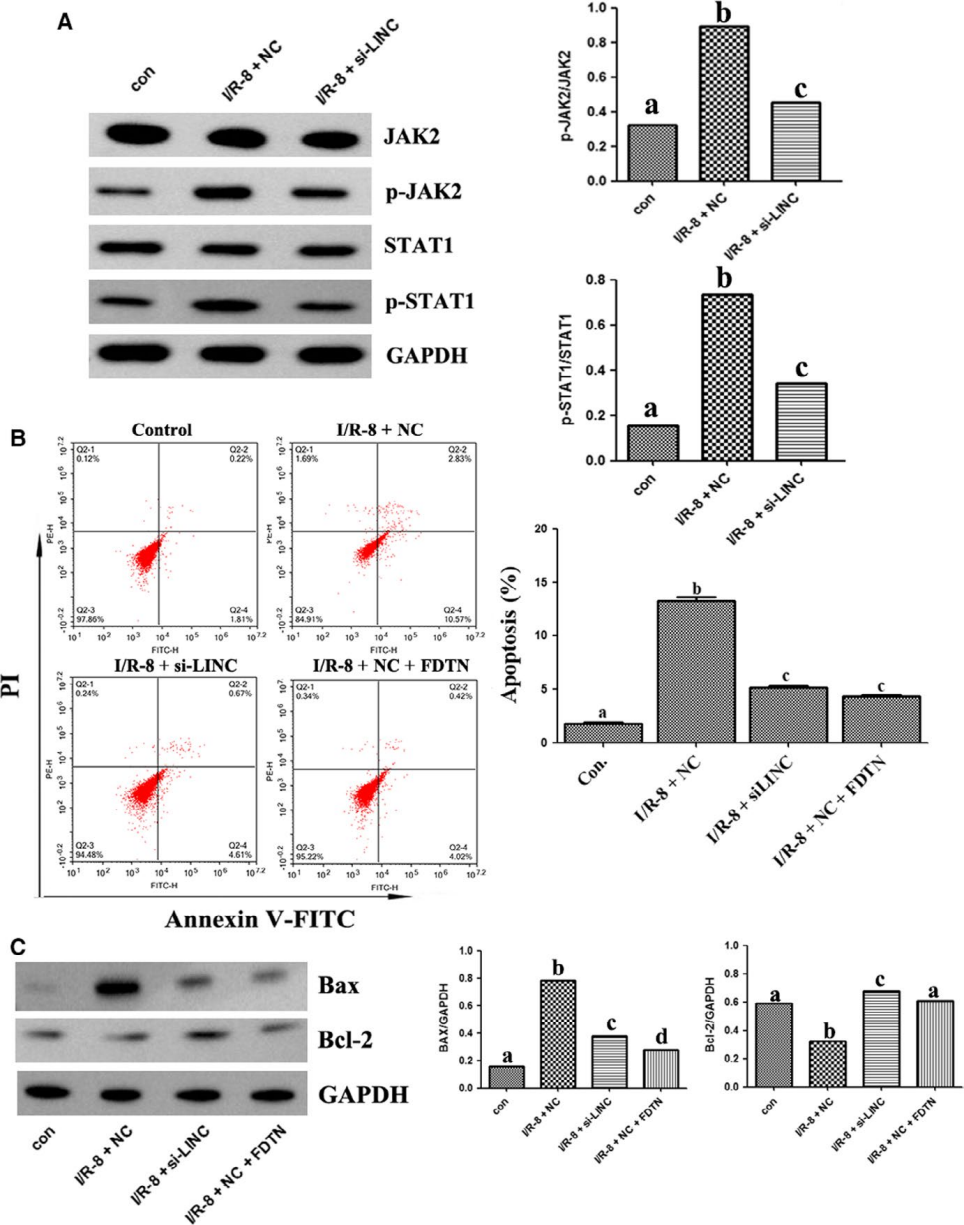
The relationship between LINC00963 and JAK2/STAT1 pathways in hypoxia‐induced HK‐2 cell model. A, The protein levels of p‐JAK2 and p‐STAT1 in different groups. B, Apoptosis analysed by flow cytometry. C, Expression of apoptosis‐related proteins by Western blot. Data are expressed as mean ± SEM, and different letters represent significant differences (*P* < .05) between groups

To verify the effect of LINC00963 on the JAK2/STAT1 signalling pathway, fedratinib (JAK2 inhibitor, FDTN) was used and HK‐2 cells were transfected with si‐LINC. In hypoxia‐induced HK‐2 cell model, Western blot showed that the expression of p‐JAK2 and p‐STAT1 was markedly reduced by transfection with si‐LINC compared with the I/R group, suggesting LINC00963 knockdown could induce the inhibition of the JAK2/STAT1 pathway (Figure 5A). Flow cytometry analyses demonstrated that knockdown of LINC00963 significantly decreased hypoxia‐induced apoptosis in HK‐2 cells, while this inhibitory effect was also been observed after down‐regulating JAK2 with FDTN (Figure 5B). Similarly, Western blot revealed that FDTN could markedly decrease Bax expression and increase Bcl‐2 expression, which exhibited a similar inhibitory effect with si‐LINC (Figure 5C).

### 3.6 LINC00963 activated JAK2/STAT1 pathway to aggravate renal I/R injury

**FIGURE 6:**
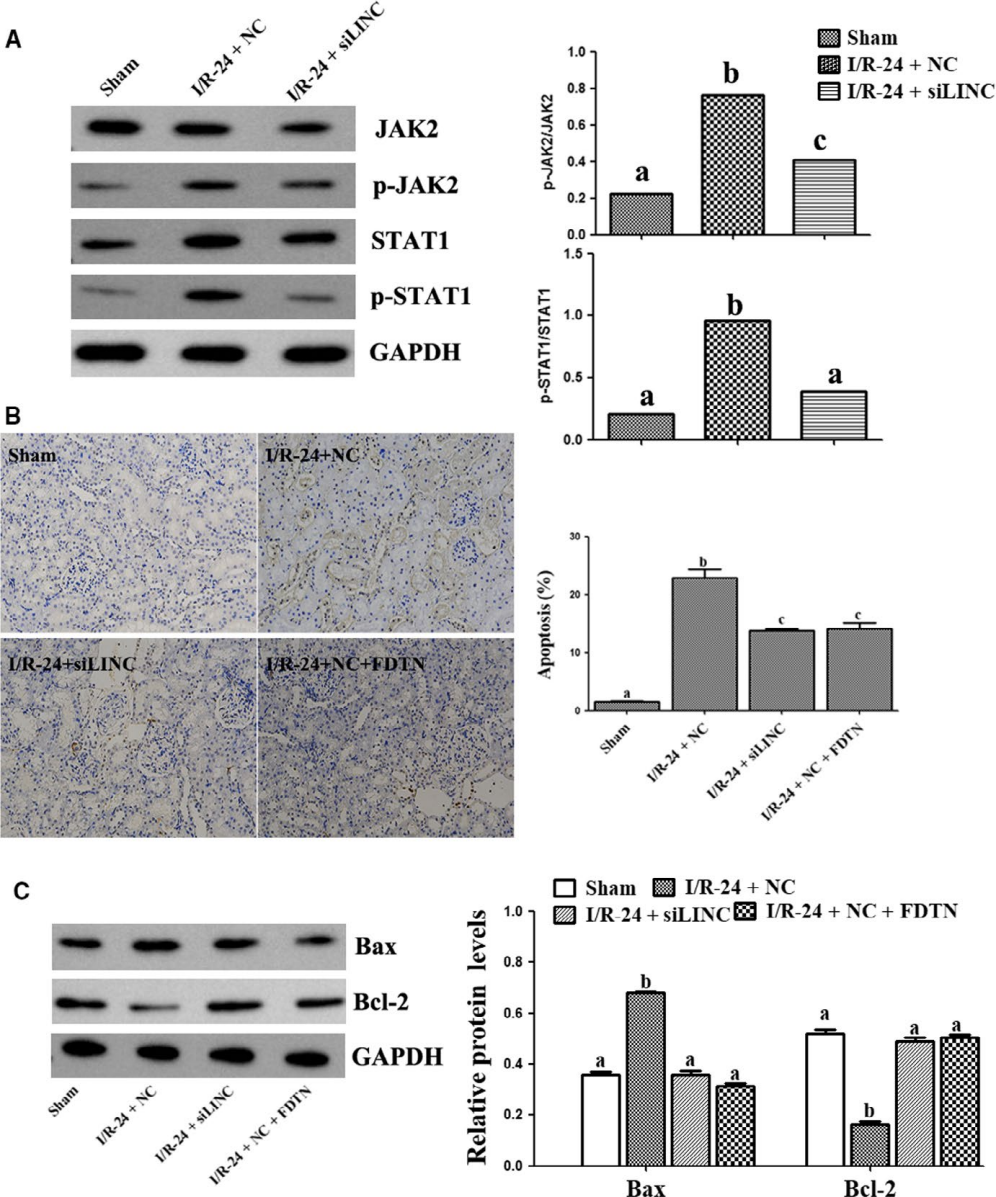
The relationship between LINC00963 and JAK2/STAT1 pathways on I/R‐induced AKI rat model. A, Expression of p‐JAK2 and p‐STAT1 under different conditions. B, TUNEL staining and cell apoptosis proportion of different groups. C, The protein levels of Bax and Bcl‐2. Data are expressed as mean ± SEM, and different letters represent significant differences (*P* < .05) between groups

The effects of LINC00963 knockdown on JAK2/STAT1 pathway in vivo were analysed by detecting the protein levels of JAK2 and its downstream protein STAT1 by Western blot. As shown in Figure 6A, compared with NC group, the expression of p‐JAK2 and p‐STAT1 significantly increased in renal tissues after transfection with si‐LINC00963. Analysis of TUNEL staining demonstrated that knockdown of LINC00963 significantly decreased I/R‐induced apoptosis in rat kidney, while this inhibitory effect slightly increased after down‐regulating JAK2 (Figure 6B). Similarly, Western blot revealed that LINC00963 knockdown markedly decreased Bax expression and increased Bcl‐2 level(Figure 6C). Together, it is concluded that LINC00963 could promote apoptosis by activating JAK2/STAT1 pathway.

## 4 DISCUSSION

Increasing evidence suggests that lncRNAs contributes greatly to kidney disease. In the field of AKI, many studies in recent years have shown that lncRNAs might play an important role.^11^, ^12^, ^13^ The expression of 2267 lncRNAs in renal tissue after I/R injury increased significantly compared with that in the normal group, and many of them were found to be involved in the pathogenesis of I/R injury according to bioinformatics analysis.^14^ Yu et al^15^ found that the expression of lncRNA PRINS in hypoxia‐induced HK‐2 cells was significantly up‐regulated and could lead to acute tubular necrosis and deterioration of renal function via normal T cell activation regulator (RANTES). The siRNA of lncRNA GAS5 could down‐regulate the mRNA and protein levels of p53 and TSP‐1, which could result in the reduction of hypoxia‐induced apoptosis of HK‐2 cells.^16^ Su et al^17^ found that renal I/R injury could up‐regulate the expression of lncRNA Malat1, which are associated with the expression of genes related to regeneration and repair of kidney tissues. Tian et al^18^ found that knockout of LINC00520 could alleviate AKI, while overexpression of LINC00520 could aggravate AKI via PI3K/AKT pathway.

As an lncRNA, LINC00963 was initially found to be associated with prostate cancer.^6^ However, LINC00963 can also be expressed in kidney tissue. LINC00963 was considered as a potential marker of the progression and outcome of chronic renal failure. Renal tissue apoptosis in chronic renal failure model of rats was found to be reduced by si‐LINC00963 through enhancing FOXO signalling pathway, which was related with renal fibrosis and oxidative stress.^7^ According to the results of our study, the expression of LINC00963 increased not only in hypoxia‐induced HK‐2 cell model, but also in I/R‐induced AKI rat model.

Most lncRNAs mainly targeted miRNA to exert their functions. To identify the target miRNA of LINC00963, RegRNA 2.0 was used for prediction in our study. Bioinformatics analyses revealed that the LINC00963 was able to accommodate multiple potential binding sites for a multitude of miRNAs. Our pull‐down and dual‐luciferase reporter assays showed miR‐128‐3p was a direct target of LINC00963 in HK‐2 cells. MiR‐128‐3p, as a tumour suppressor, was previously identified to be down‐regulated in hepatocellular carcinoma tissues and cell lines.^19^ Recent study suggested that miR‐128‐3p was related to the cell proliferation and apoptosis.^20^ Increasing evidences suggested that dysregulated miRNAs were involved in the pathogenesis of AKI.^21^ For instance, inhibition of miR‐182 improved kidney function and morphology after AKI.^22^ MiR‐204 protected interstitial tissue of renal tubules from fibrotic change after AKI induced by ischaemia‐reperfusion.^23^ Effect of miR‐128‐3p on the apoptosis of hypoxia‐induced HK‐2 cells was studied in our experiments, which indicated that inhibition of miR‐128‐3p could reversely increase the apoptosis of HK‐2 cells silenced by si‐LINC00963.

JAK2/STAT1 pathway is a key molecular pathway for variety of cytokines and growth factors to transmit signals in cells and participates in biological activities such as immune response, vascular cell migration and apoptosis.^24^ Several studies have demonstrated the contribution of JAK/STAT pathway to the I/R injury of various organs.^25^, ^26^, ^27^ Study had showed that the inhibition of JAK2/STAT1 pathway could decrease renal tissue apoptosis in cisplatin‐induced AKI.^28^ Our results suggested that phosphorylation of JAK2/STAT1 pathway, which can be regulated by si‐LINC00963, could promote apoptosis of hypoxia‐induced HK‐2 cells and I/R injury renal tissues.

In conclusion, LINC00963 was increasingly expressed in AKI renal tissue. Blocking LINC00963 could target miR‐128‐3p to reduce the apoptosis of renal tubular epithelial cell through JAK2/STAT1 pathway to hinder the progression of AKI.

## CONFLICT OF INTEREST

The authors declare that they have no competing interests.

## AUTHORS' CONTRIBUTION

LBX and RJ designed the study; BC, XL, YFC, RY, SRH and LJP performed the experiments; LBX and BC collected and analysed the data. LBX wrote the manuscript. All authors read and approved the final manuscript.

## Data Availability

The data that support the findings of this study are available from the first author upon reasonable request.
